# Functional Responses of Salt Marsh Microbial Communities to Long-Term Nutrient Enrichment

**DOI:** 10.1128/AEM.03990-15

**Published:** 2016-04-18

**Authors:** Christopher J. Graves, Elizabeth J. Makrides, Victor T. Schmidt, Anne E. Giblin, Zoe G. Cardon, David M. Rand

**Affiliations:** a Brown University, Department of Ecology and Evolutionary Biology, Providence, Rhode Island, USA; b Brown University, Division of Applied Mathematics, Providence, Rhode Island, USA; c Marine Biological Laboratory, Josephine Bay Paul Center for Comparative Molecular Biology and Evolution, Woods Hole, Massachusetts, USA; d Marine Biological Laboratory, Ecosystems Center, Woods Hole, Massachusetts, USA; University of Calgary

## Abstract

Environmental nutrient enrichment from human agricultural and waste runoff could cause changes to microbial communities that allow them to capitalize on newly available resources. Currently, the response of microbial communities to nutrient enrichment remains poorly understood, and, while some studies have shown no clear changes in community composition in response to heavy nutrient loading, others targeting specific genes have demonstrated clear impacts. In this study, we compared functional metagenomic profiles from sediment samples taken along two salt marsh creeks, one of which was exposed for more than 40 years to treated sewage effluent at its head. We identified strong and consistent increases in the relative abundance of microbial genes related to each of the biochemical steps in the denitrification pathway at enriched sites. Despite fine-scale local increases in the abundance of denitrification-related genes, the overall community structures based on broadly defined functional groups and taxonomic annotations were similar and varied with other environmental factors, such as salinity, which were common to both creeks. Homology-based taxonomic assignments of nitrous oxide reductase sequences in our data show that increases are spread over a broad taxonomic range, thus limiting detection from taxonomic data alone. Together, these results illustrate a functionally targeted yet taxonomically broad response of microbial communities to anthropogenic nutrient loading, indicating some resolution to the apparently conflicting results of existing studies on the impacts of nutrient loading in sediment communities.

**IMPORTANCE** In this study, we used environmental metagenomics to assess the response of microbial communities in estuarine sediments to long-term, nutrient-rich sewage effluent exposure. Unlike previous studies, which have mainly characterized communities based on taxonomic data or primer-based amplification of specific target genes, our whole-genome metagenomics approach allowed an unbiased assessment of the abundance of denitrification-related genes across the entire community. We identified strong and consistent increases in the relative abundance of gene sequences related to denitrification pathways across a broad phylogenetic range at sites exposed to long-term nutrient addition. While further work is needed to determine the consequences of these community responses in regulating environmental nutrient cycles, the increased abundance of bacteria harboring denitrification genes suggests that such processes may be locally upregulated. In addition, our results illustrate how whole-genome metagenomics combined with targeted hypothesis testing can reveal fine-scale responses of microbial communities to environmental disturbance.

## INTRODUCTION

Environmental nutrient addition represents a pervasive disturbance to coastal ecosystems. In estuarine systems, large-scale anthropogenic nutrient addition can profoundly alter ecosystem processes, leading to declining water quality, hypoxia, and blooms of undesirable algae (1–3). While there have been a large number of studies documenting how specific microbial processes have changed as a result of nutrient inputs and other disturbances (4–6), relatively few studies have examined functional changes due to nutrient loading across the entire microbial community. Of particular interest for remediation of salt marshes is the microbial capacity to transform biologically active pollutant nitrogen compounds to nitrogen gas via denitrification—a metabolic process shared by many taxonomically diverse bacteria (7). The extent to which microbial denitrification is altered by bacterial responses to nutrient addition will ultimately determine the amount of nitrogen making its way into marine systems and could thereby influence the development of hypoxic zones and the emission of potent greenhouse gases, such as nitrous oxide.

Studies of microbial community responses to anthropogenic nutrient addition have yielded mixed results. Many find no clear changes in community composition, even in the face of heavy nutrient loading accompanied by obvious macroscale environmental change (8–10). This led to early suggestions that microbial communities are resistant to nutrient loading and other disturbances (10). However, targeted approaches examining the response of specific genes related to nutrient metabolism suggest that fine-scale community responses occur despite overall similarity in community composition. For example, in-depth sequencing of the nitrite reductase gene, *nirS*, by Bowen et al. (11), recently showed increases in the abundance of *nirS* at nitrate-enriched sites which had previously shown a lack of differentiation based on overall taxonomic community composition. Indeed, a wide range of studies have shown responses by specific genes or groups of genes to a given environmental variable, including the impact of fire (6), antibiotics (12), soil cultivation (13), and diet (14). Furthermore, when microbial functionality is measured directly, the functional capacity of soil microbial communities is directly influenced by nutrient loading (15, 16). Together, these studies suggest that taxonomic comparisons of the total microbial community may lack the resolution necessary to reveal fine-scale functional responses.

In this study, we analyzed the effect of long-term nutrient addition on sediment microbial communities in a New England salt marsh estuary using whole-genome metagenomics. We compared these metagenomic profiles along a reference creek and a creek exposed to long-term sewage effluent runoff in which nitrate and other nutrients have been released into the environment for over 4 decades. We compared the communities at 3 levels. First, we compared summary metrics of metagenomic annotations of the whole data set. Next, we analyzed subsets of the data set corresponding to broad gene categories that we hypothesized would respond to nutrient addition, and we performed agnostic searches for gene categories with highly divergent distributions between creeks. Finally, we explored the relative abundance and taxonomic distribution of a single gene, that encoding nitrous oxide reductase (*nosZ*), which is associated with the denitrification process. We analyzed both the typical and a recently recognized atypical variant of *nosZ* (17–19) across a wide range of bacterial taxa, and we compared their distributions between creeks.

Many common nitrogen transformations can be carried out by a wide range of bacterial taxa (20, 21); therefore, community responses to nutrient addition are likely to result from small changes among metabolically redundant taxa rather than from large changes in a specific group. Thus, we hypothesized that taxonomic metrics of whole communities would be similar between the creeks, as observed in previous studies (8–10), but that genes coding for enzymes involved in dissimilatory nitrate reduction, such as those involved in denitrification, would be found at higher frequencies in response to continual anthropogenic nutrient input.

## MATERIALS AND METHODS

### Study site.

**FIG 1:**
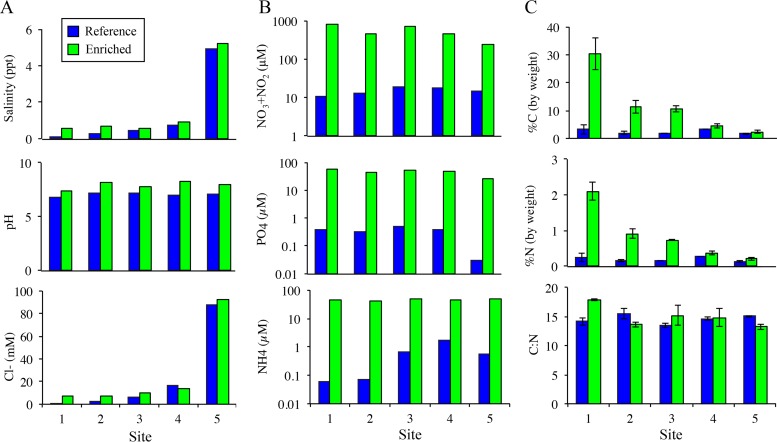
Physical characteristics of water and sediment from the reference and enriched creeks. A single water sample was collected at each site (A and B), while three sediment samples were collected per site (C). (A) Similar gradients between the two creeks from sites 1 through 5 in aspects unrelated to sewage outfall. (B) The sewage outfall is associated with significant nutrient enrichment, with orders-of-magnitude greater concentrations of nitrate/nitrite, phosphorus, and ammonia in water at all sites along the gradient. (C) Concentration of total carbon and nitrogen content in creek sediment are significantly higher in the enriched creek than in the reference creek (*P* < 0.01).

Two creeks located in Ipswich, MA, were chosen for this study, Greenwood and Egypt Creeks, here referred to as the enriched and reference creeks, respectively. Both creeks flowed into Plum Island Sound, were regularly fed by freshwater terrestrial streams, and experienced daily tidal height changes of 3 to 4 m. The two creeks were located within 5 km of one another, were surrounded by similar salt marsh plant communities, and had similar salinity gradients (Fig. 1; see also Fig. S1 in the supplemental material). However, the two creeks differed dramatically in the water quality of the freshwater input, with the enriched creek receiving input from Ipswich sewage effluent located near the head of the creek and the reference creek receiving input from a local drinking water reservoir. The treated sewage effluent in the enriched creek came from a wastewater treatment plant that has been in operation since 1958 and that UV-sterilizes wastewater before releasing it into the salt marsh. A satellite image showing the relative locations of the two creeks and the sampling sites within is shown in Fig. S1 in the supplemental material.

### Sampling design.

Sediments were sampled within a 2-h period during low tide on 8 October 2011. Five sites were chosen in an ∼2-km stretch along each creek (see Fig. S1 in the supplemental material), with the sites tracking a similar salinity gradient (Fig. 1). Our upstream sampling sites were located within 30 m of the head of each creek, which was less than 10 m from the sewage effluent in the enriched creek (see Fig. S1). Salinity was measured on site with YSI 85 (Yellow Springs, OH) instrument. Sediment samples were taken from the low intertidal portion of the creek by taking 2-cm cores using sterile 50-ml polystyrene tubes. At each sampling site, three subsites were chosen within 2 m of one another for sediment sampling. At each subsite, 3 sediment cores were collected close to one another and thoroughly mixed to minimize fine-scale heterogeneity. Thus, there were 3 sediment samples from each site for metagenomic analysis.

The sediment samples were stored on ice until they could be taken out of the field for further processing. Once in the lab, large visible pieces of plant material were removed, and the sediment samples were aliquoted into 2-ml tubes, flash-frozen in liquid N, and stored at −80°C. One additional sediment core was taken at each sampling site for analysis of the physical and chemical properties as was a 50-ml sample of water from the creek, which was filter-sterilized through a 0.2-μm polycarbonate filter.

### Analysis of water and sediment chemistry.

Water samples were used to quantify salinity as well as pH and nutrients ([NO_3_^−^ + NO_2_^−^], [PO_4_^−^], [NH_4_^+^], [Cl^−^], and [SO_4_^−^]). The additional sediment sample was used to measure gravimetric moisture content, % C, and % N by weight. In the lab, salinity of water samples was measured by determining specific conductivity using a CDM 210-m radiometer analytical probe (platinum electrode, 2 poles). The pH of each water sample was determined with an Orion 520A meter with a Ross combination pH electrode. The PO_4_ concentration in the water was measured using the spectrophotometric method, as described by Murphy and Riley (22). The combined concentration of nitrate and nitrite in the water was determined using the cadmium reduction method on a flow injection analyzer (Lachat QuikChem 8000). The ammonium concentration in the water was measured by the phenol hypochlorite method, as described by Solorzano (23). Chorine and sulfate concentrations were measured using a Dionex ion chromatograph (ThermoFisher Scientific, Pittsburgh, PA).

### Metagenomic sequencing.

Genomic DNA was extracted from sediment using the MoBio PowerSoil kit (Carlsbad, CA), and concentration and quality of the DNA preparations were determined using a Nanodrop spectrophotometer (ThermoFisher). The purified genomic DNA was sheared to 150 to 200 bp using the Covaris S220 (Woburn, MA) acoustic system. Metagenomic libraries were prepared using the NuGen Encore multiplex systems I and IB (NuGen, Carlsbad, CA). The manufacturer's instructions were followed for end repair, adapter ligation, amplification, and barcoding, with unique DNA barcodes flanking the adapters for each sample and replicate. The distribution of insert sizes was confirmed using an RNA picochip (Agilent, Santa Clara, CA), and concentrations of the libraries were measured using qPCR. Equal amounts of barcoded DNA from each replicate and sampling site were used for a 100-bp paired-end run on two Illumina HiSeq 2000 flow cell lanes. Sequencing was conducted at the Brown University Illumina core facility, Providence, RI, and at the marine biological laboratory, Keck sequencing facility, Woods Hole, MA.

### Metagenomic sequence annotation.

Paired-end reads for each sample were joined using FastqJoin (24), with a minimum overlap of 8 bp and a maximum 10% discrepancy. Paired reads that did not overlap at the required level were omitted from subsequent analyses. Joined sequences were quality filtered using DynamicTrim (25), with a minimum phred score of 15 and a maximum of 5 base calls below this score. Resulting sequences were submitted to the MG-RAST server (26) for annotation. Annotations were created for each sequence using the KEGG database, with a maximum E value cutoff of 1e−5, minimum percent identity cutoff of 60%, and minimum alignment length of 30 nucleotides (nt). Across all samples, approximately 250,000 unique annotations were identified, with each sample having between 50,000 and 75,000 distinct annotations. Annotation frequencies within each sample were calculated by normalizing individual annotation counts to total annotation counts for that sample, excluding a single poorly characterized annotation for “hypothetical protein,” which was inexplicably found at high abundance in only some of our samples. Removal of this annotation did not qualitatively affect the reported results.

### Multivariate analyses of community composition.

Principal-component analyses (PCA) were conducted to summarize relationships between our samples with respect to functional and taxonomic compositions. These analyses were conducted using MG-RAST genus-level taxonomic annotations of sequence reads and MG-RAST functional subsystem annotations using PRIMERe v6.1.12.

### Targeted annotation analysis.

KEGG annotations of our metagenomic sequences were first analyzed using a targeted approach based on specific biogeochemical denitrification annotations that we hypothesized would respond to nitrogen loading. We used string-based word searches in Matlab (Mathworks, Natick, MA) to identify annotations corresponding to genes and enzymes involved in steps of denitrification, specifically respiratory nitrate reductase (e.g., *narG*, *napA*), dissimilatory nitrite reductase (e.g., *nirK* and *nirS*), and nitrous oxide reductase (e.g., *nosZ*) functions. These divisions are by no means an exhaustive exploration of all processes that may be influenced by nutrient loading but, rather, represent only a few processes which we hypothesized *a priori* would respond to nutrient loading. Since anaerobic, heterotrophic denitrification is expected to be particularly stimulated by the higher nitrate concentrations in creek water or possibly by an increase in labile organic matter in the sediments, we chose genes involved in this process for investigation. The string-based search strategy identifies sequences coding for the target enzymes themselves as well as for other known regulatory genes (e.g., within operons) associated with those target enzymes.

### Effects of nutrient input on functional gene abundance.

Once annotation groups were defined, we calculated the frequency of each group at each site across enriched and reference creeks and tested our hypothesis that nutrient loading increased their frequencies in the enriched creek using nonparametric permutational multivariate analysis of variance (MANOVA) models (27). The models included both creek (reference or enriched) and bulk sediment carbon percentages (Fig. 1C) as factors and were run over Bray-Curtis similarity matrices for each annotation subgroup. These analyses were conducted in the R statistical programming language (v. 3.1.2) using the adonis implementation in the vegan community ecology library (28), using 10,000 permutations within each factor. These analyses provided a robust method to compare the relative contributions of nutrient input in driving differences in annotation frequencies between the creeks relative to that of natural variation between the creeks.

### Agnostic search of diverging annotations.

To assess the robustness of our targeted hypothesis testing of annotation groups involved in denitrification, an agnostic string-based search across the entire data set was used to identify highly divergent annotations between the two creeks. This approach makes no *a priori* assumptions about which annotations may respond to nutrient loading but instead seeks to identify highly divergent groups of annotations between the two creeks. Importantly, this approach was used only to determine whether the divergence of annotations related to denitrification could be recovered with an agnostic data mining approach and to identify possible functional categories warranting further analysis. To accomplish this, we parsed all annotations into component word strings separated by spaces or the following symbols: en dash (–), single quotation (′), double quotation (“), parenthesis pair, bracket pair, hyphen (-), slash (/), or semicolon (;). Then, we grouped all identical words, along with their corresponding annotations, into a word group. For example, the annotations “nitric-oxide reductase” and “nitric oxide reductase” would both belong to the word groups “nitric,” “oxide,” and “reductase.” The colon (:) and period (.) symbols were not used to separate words, so Enzyme Commission numbers were maintained as complete words (when present).

Once the annotations were parsed into word groups, we performed searches for annotation groups that were highly divergent between the two creeks. We accomplished this by looking for subsets of annotations within a given word group that had highly divergent frequencies across the two creeks, and we examined whether the higher frequencies occurred in the reference or enriched creek. To do this, we used a Wilcoxon rank sum test procedure to generate a metric with which we grouped annotations, using frequency at each site as the ranked variable and creeks as the Wilcoxon categories. We subsequently grouped annotations based on the *P* value of the rank test: effect level 3 (*P* < 0.001), effect level 2 (*P* < 0.01), effect level 1 (*P* < 0.05), and effect level 0 (all annotations). We emphasize that these Wilcoxon rank-sum tests were used only to create effect level groupings and were not intended to establish significant differences between creeks for any single annotation (this effort would be impeded by the many multiple comparisons). We further assigned each grouping a score between −1 and +1 according to whether higher frequencies occur in the reference or enriched creek. Specifically, we computed an annotation ratio as follows: annotation ratio = (HE − HR)/(HE + HR), where HE is the number of annotations within a given grouping with a higher median frequency in the enriched creek, and HR is the number of annotations in that same grouping with a higher median frequency in the reference. Thus, an annotation ratio of +1 indicated that every annotation within a grouping had a higher median frequency in the enriched creek, while a value of −1 indicated that every annotation had a higher median frequency in the reference creek.

To identify highly divergent word groups across the two creeks, we searched for those with relatively large numbers of annotations in effect level 3, and with a strong and consistent directionality in the frequency differences, as captured by the annotation ratio. In particular, we identified word groups meeting the following three criteria: first, for each of the effect level 1, effect level 2, and effect level 3 bins, the absolute value of the annotation ratio was greater than 0.5; second, the ratio of the number of annotations in the effect level 3 bin to the total number of annotations in the word group was at least 500% greater than the comparable ratio for the entire data set; and third, the word group contained more than 10 annotations. Word groups meeting these criteria totaled approximately 250 out of 70,000 possible groups. We note that we do not make any claims with respect to statistical significance but, rather, posit that these groups represent interesting targets for further study.

### NosZ bioinformatic analysis.

In addition to groupings of annotations associated with broad steps in the denitrification pathway, some of which are accomplished with multiple genes, we also focused more specifically on *nosZ*, the gene coding for nitrous oxide reductase. To explore the influence on nitrogen loading on the frequency of recently described typical and atypical forms of *nosZ*, quality-filtered metagenomic reads were queried against a reference set of NosZ amino acid sequences. This reference set was built by downloading all amino acid sequences from the NCBI protein database matching the search query “nosz” on 2 December 2013. Any identical sequences, sequences less than 600 amino acids in length, or sequences corresponding to other genes in the *nos* operon were removed from the reference set. Each sequence in the curated *nosZ* reference set was then classified as typical or atypical based on whether it belonged to a genus categorized as having typical or atypical *nosZ*, as in the report by Sanford et al. (17). Sequences which could not be categorized with this approach were excluded from the analysis, leaving a total of 169 typical *nosZ* sequences and 70 atypical sequences. Metagenomic reads were then queried against this database, with all six reading frame translations of each read using tblastn implemented in the SWIPE aligner (29) and with a bit score threshold of 80.

Some of our metagenomic reads had multiple matches to both typical and atypical sequences in the *nosZ* reference set, with a bit score greater than 80. A weighted bootstrapping approach with 1,000 resampling events was used to assign confidence intervals to the frequency of atypical versus typical *nosZ* sequences estimated in each site. During each of the resampling events, one of the matching reference sequences was chosen for each read in proportion to its bit score. The bootstrap distribution generated with this approach was used to assign 95% confidence intervals to the frequency of atypical metagenomic reads in each creek.

## RESULTS

### Strong nutrient load in the enriched creek.

The sewage effluent at the head of the enriched creek resulted in strong nutrient gradients in the water and sediment compared to the reference creek, which had an influx of clean freshwater (Fig. 1). While salinity, pH, chloride, and sulfate concentrations were similar in water samples taken at corresponding sites in the two creeks (Fig. 1A; see also Data set S1 in the supplemental material), concentrations of nitrate/nitrite, ammonium, and phosphate in water were far greater throughout sites in the enriched creek (Fig. 1B; see also Data set S1). We also found a strong gradient in the carbon and nitrogen contents of the enriched creek, with the highest levels observed in sediment samples near the head of the creek (Fig. 1C; see also Data set S1). In contrast, nutrient contents were similar and comparatively low among all of the sites in the reference creek, and we did not see a strong gradient in sediment carbon or nitrogen concentration. The sewage effluent had received secondary treatment, so the strong gradient in sediment carbon most likely reflected increases in primary production close to the outfall due to nutrient additions rather than a direct input of carbon from the outfall, though we recognize that without direct samples of effluent prior to entrance into the creek, we cannot state this with certainty.

### Metagenomic annotation summary data.

Metagenomic sequencing of 3 replicates at 5 sites along each of the creeks resulted in 30 samples, which were sequenced to an average depth of 8.16 × 10^6^ reads (standard error, ±3.4 × 10^5^) after quality filtering. Annotation in MG-RAST mapped between 24% and 42% of the reads to known proteins, yielding 3.1 × 10^6^ (±1.5 × 10^5^) protein annotations per replicate.

**FIG 2:**
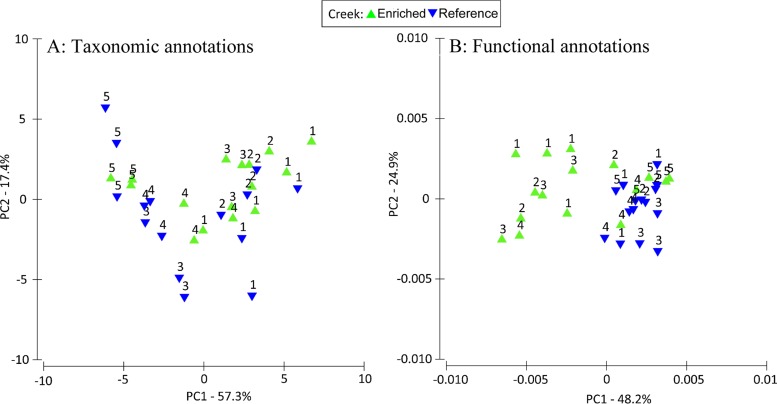
Principal-component analysis of genus-level taxonomic annotations (A) and subsystems-level functional annotations (B) between each site and creek.

Taxonomic annotations showed great similarity in composition between the two creeks, with a high degree of concordance in the frequencies of the most abundant genera (see Fig. S2A in the supplemental material). In both creeks, Geobacter, Bacteroides, Pseudomonas, Desulfovibrio, and Burkholderia were among the most abundant genera. Furthermore, PCA plots based on class-, order-, and genus-level classifications all revealed similar patterns, with the sample site along the creek separating samples along principal component axis 1 (PC1) but with no clear separation by creek type (Fig. 2A). Both creeks also showed similarities based on PCA analysis of KEGG functional subsystems (Fig. 2B), with identical rankings of the top 10 most abundant subsystems (see Fig. S2B in the supplemental material). In contrast, a PCA plot of functional subsystems data did indicate some separation along PC1 by creek, particularly at site 13, which is the closest to the outfall (Fig. 2B).

### Increased frequency of denitrification-associated annotations in the enriched creek.

**FIG 3:**
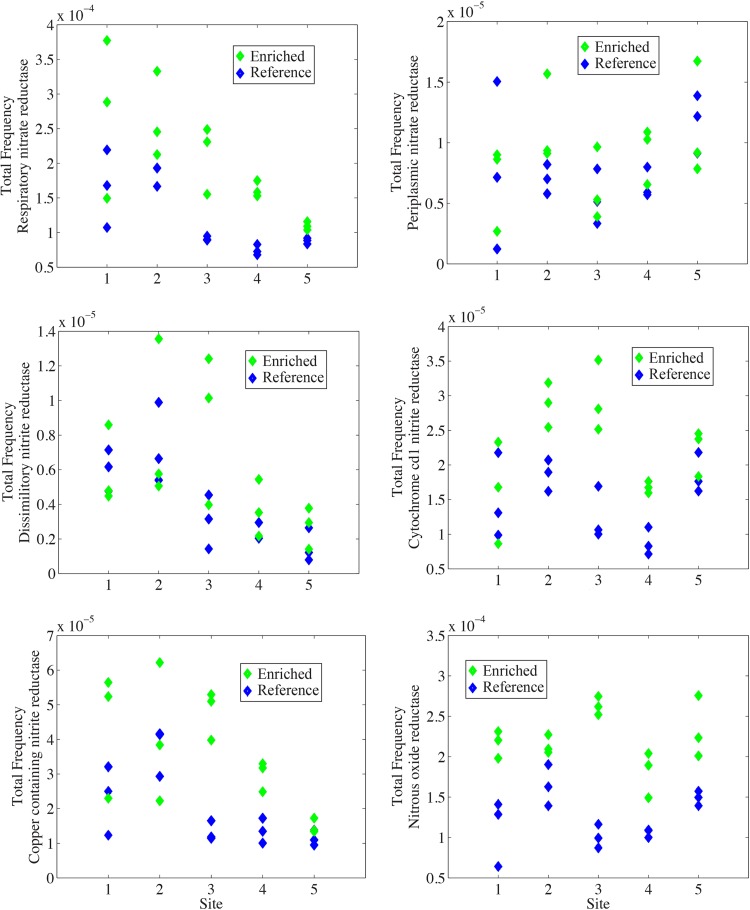
Plots showing the frequency of annotations corresponding to enzymes associated with various steps in the denitrification pathway. Each panel represents the abundance of annotations corresponding to a given enzyme across all 5 sites in both the enriched and reference creeks. Multiple points at each site represent replicate samples.

We hypothesized that increased concentrations of nitrate in the enriched creek would lead to increases in denitrifying bacteria and, correspondingly, to increases in the frequency of annotations linked to denitrification. Annotations that were labeled as nitrate reductase, nitrite reductase, nitrous oxide reductase, and nitric oxide reductase were strongly increased in sites from the enriched creek compared to the reference creek (see Fig. S3 in the supplemental material). Since these broadly defined groups likely included some annotations unrelated to denitrification, we also focused on subsets of annotations that corresponded to more specific steps in the denitrification pathway. Among them, annotations associated with respiratory nitrate reductase, copper-containing nitrite reductase, cytochrome *cd*_1_ nitrite reductase, nitric oxide reductase, and nitrous oxide reductase showed significantly higher frequencies in the enriched creek than in the reference creek (Table 1; Fig. 3). Importantly, both nutrient concentration (% C) and creek were significant factors in most of our analyses, demonstrating that differences in annotation abundances between the creeks are at least partly related to nutrient enrichment from the sewage effluent (Table 1).

**TABLE 1:** Annotation subgroups, defining search criteria of subgroups, total number of annotations found in each group, and *P* values from permutational analysis of variance tests for each annotation subgroup

Annotation subgroup	Search criteria	No. of annotations	*P* value for^*a*^:		
			Creek effect	% C effect	Creek × % C interaction effect
Respiratory nitrate reductase	“respiratory” AND “nitrate reductase”	87	**0.0023**	**0.0001**	0.3348
Assimilatory nitrate reductase	“assimilatory” AND “nitrate reductase”	32	**0.0412**	**0.0047**	0.1677
Periplasmic nitrate reductase	“periplasmic” AND “nitrate reductase”	62	**0.0017**	0.2235	0.1746
Dissimilatory nitrite reductase	“dissimilatory” AND “nitrite reductase”	7	0.2893	**0.0194**	0.8480
Cytochrome *cd*_1_ nitrite reductase	“cytochrome cd_1_” AND “nitrite reductase”	10	**0.0023**	0.0710	0.7731
Cytochrome *c* nitrite reductase	“cyctochrome c” AND “nitrite reductase”	18	**0.0171**	0.2551	0.2249
Assimilatory nitrite reductase	“assimilatory” AND “nitrate reductase”	32	0.1173	**0.0061**	0.1289
Nitric oxide reductase	“nitric oxide reductase”	128	**0.0027**	**0.0145**	0.1510
Nitrous oxide reductase	“nitrous oxide reductase” OR “*nosX*” (where X = any letter)	102	**0.0001**	**0.0272**	0.5109

a % C, % carbon. Significant results after Bonferroni correction are indicated in bold.

Last, our data mining approach used to agnostically identify string-based annotation groups that differ in abundance between the two creeks recovered multiple groups related to the nitrogen cycle, including all broad annotation groups from our targeted enzymes in Table 1 (see also Fig. S3 in the supplemental material). Among the annotation groups identified with this approach, those related to denitrification were recovered among those differing most strongly and consistently in abundance between the two creeks; among annotation groups with at least 100 members, 7 of the top 11 most divergent were related to denitrification (see Data set S3 in the supplemental material). This approach also identified several unrelated functional groups that greatly differed between the two creeks. These included collections of genes related to bacterial conjugation and horizontal gene transfer; various components of bacterial defenses, particularly components of the clustered regularly interspaced short palindromic repeat (CRISPR)/Cas prokaryotic viral defense mechanism, as well as known bacteriophages; genes related to heavy metal resistance; and genes for variant strategies of protection from osmotic stress (see Fig. S4 in the supplemental material).

### Broad taxonomic response of *nosZ* to nutrient loading.

**FIG 4:**
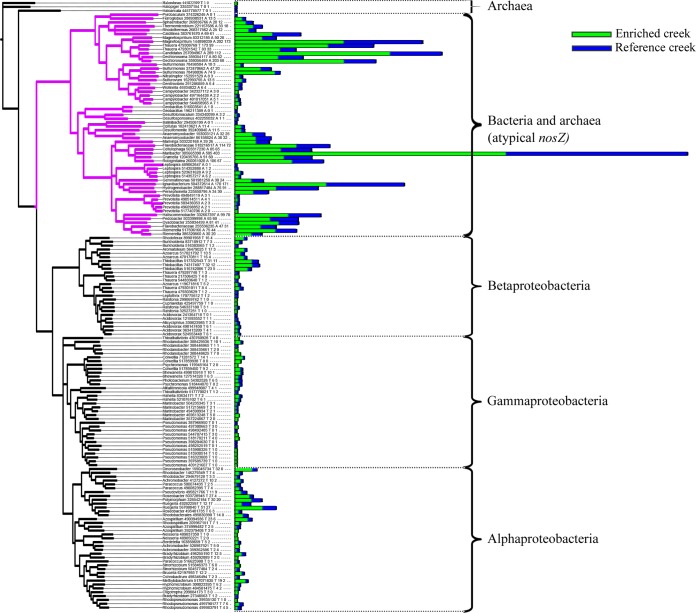
Bootstrapped neighbor-joining phylogeny of 158 *nosZ* sequence clusters from our original 240-reference NCBI-based database, representing 80 distinct bacterial and 3 archaeal genera. Clusters of >93% similarities were formed to reduce the total spread of the tree using UCLUST (47). Sequences in this tree had at least 1 hit from our creek sediment metagenomic data with an alignment bit score greater than 80. The number of hits from enriched and reference creeks to each cluster is shown by the length of the bar at the terminal node of each branch. Branches are colored by typical (gray) versus atypical (violet) genera according to categorization of atypical and typical genera by Sanford et al. in 2012 (17).

Our metagenomic analyses revealed dramatic differences in the frequency of nitrous oxide reductase (*nosZ*) between the two creeks, with substantially greater frequencies found in the enriched versus the reference creek (Table 1; Fig. 3). The frequency of sequences from our metagenomic libraries with high-quality hits to our curated set of *nosZ* reference sequences was again higher in the enriched creek (2.4 × 10^−5^) (3,875 total reads) than in the reference creek (1.2 × 10^−5^) (2,355 total reads), confirming our conclusions based on MG-RAST–generated KEGG annotations that the gene is more prevalent in the enriched creek. The taxonomic distributions of *nosZ* were similar between the two creeks (Fig. 4; see also Fig. S5 in the supplemental material), suggesting that the increased abundance of *nosZ* was spread over a broad taxonomic distribution of *nosZ*-harboring microorganisms.

Our approach also allowed us to assess the environmental frequency of a recently characterized *nosZ* variant termed atypical *nosZ* (17). Interestingly, both creeks showed a higher proportion of ayptical *nosZ*, which represented 72.85% (95% CI, 72.35% to 73.34%) in the enriched creek and 79.99% (95% CI, 79.39% to 80.56%) in the reference creek (Fig. 4; see also Fig. S5 in the supplemental material). Last, our metagenomic reads mapped to 83 distinct genera from our reference set, with bit scores greater than 80.

## DISCUSSION

We measured the response of salt marsh microbial communities to long-term nutrient enrichment using whole-genome metagenomic analysis of two creeks, one which has been enriched by sewage effluent at its headwaters for over 40 years and a second, reference creek fed by clean water from a local water supply. We found that the physical and chemical properties of the water and sediment were well matched in the enriched and reference creeks at corresponding sampling locations, with similar pH, temperature, and salinity levels in the water (Fig. 1A). Yet, concentrations of nitrate, ammonium, and phosphate were substantially higher in the enriched creek, which had been exposed to sewage effluent for over 40 years (Fig. 1B); bulk nitrogen and carbon content in the sediment samples also were substantially higher in the enriched creek (Fig. 1C). While nutrient input to the creek from sources other than the sewage effluent is possible, the long-term and consistent presence of a sewage outfall in an otherwise undeveloped area (see Fig. S1 in the supplemental material) is likely the primary driver of this nutrient gradient. Functional responses of microbial communities to this nutrient enrichment were modest when all functional annotations were analyzed in aggregate (Fig. 2; see also Fig. S2 in the supplemental material), potentially reflecting the broadly similar abiotic characteristics of the two habitats. However, metagenomics reads matching enzymes across the denitrification process were significantly more abundant in the enriched creek, suggesting a targeted functional response of sediment communities to nutrient enrichment (Fig. 3; Table 1). This increase in the abundance of denitrification genes continued at sampling sites far downstream of the sewage effluent (Fig. 3), suggesting that nutrient input in the headwaters had strong effects throughout the length of the creek. Bulk carbon content (% C) was a significant factor explaining differences between the creeks; however, % C alone did not account for differences along the length of the enriched creek. This is likely because our low-tide sampling underestimated the strength of the gradient and because sediment carbon is another factor driving denitrification. We did find that % C in sediment along the creeks was a significant factor explaining variation in the frequency of denitrification genes, suggesting that these differences are not solely attributable to natural variation between the two creeks studied (Table 1). Further, this overall pattern was robust against inclusion of either % C or % N as a proxy for the level of nutrient enrichment at our sites. Taxonomic assignments of metagenomic reads mapping to nitrous oxide reductase (*nosZ*) demonstrated that increases in the frequency of this gene were distributed across a broad taxonomic range rather than confined to a particular group of related bacteria. Together, these data support a taxonomically broad but functionally targeted response of microbial communities to long-term nutrient enrichment.

Previous studies of microbial community responses to anthropogenic nutrient addition have yielded mixed results. Many find no clear changes in taxonomic community composition by using 16S rRNA-based approaches, even in the face of heavy nutrient loading accompanied by obvious macroscale environmental change (8–10). This led to early suggestions that microbial communities are resistant to nutrient loading and other disturbances (10). However, the redundancy in metabolic functions between diverse bacterial groups and the ease with which bacteria can acquire new metabolic functions through horizontal gene transfer (30, 31) may make marker genes that are not functionally related to the anthropogenic disturbance (e.g., 16S rRNA) unsuitable for detecting the type of fine-scale community responses that we revealed here. Our study adds to the growing literature which suggests that a disconnect often exists between taxonomy and function which limits the ability of taxonomy-based approaches to further our understanding of the functional changes in a community (32, 33).

### Broad-scale changes to communities after long-term nutrient enrichment.

Community-level analyses of the functional composition of bacteria in our samples show overall similarity of the two creeks; however, there are detectable variations in functional and taxonomic annotations along each creek (Fig. 2). Free-living microbial communities respond strongly to environmental salinity (34, 35), providing an intuitive explanation for taxonomic separation by sites along the salinity gradient (Fig. 1A) in our PCA of taxonomic data (Fig. 2A). Interestingly, a similar effect of creek site was not observed during PCA of functional subsystems data (Fig. 2B). Instead, sites closest to the sewage effluent in the enriched creek separate along the first principal component, suggesting some separation in functional annotations in response to the sewage effluent (Fig. 2B). It is likely that metagenomic sequence annotations do not have the taxonomic resolution necessary to detect creek-level effects in our samples, and disagreement exists regarding the reliability of taxonomic assignments from metagenomic annotations (36). However, the observation of clear gradient effects on taxonomic composition, even in the absence of explicit environmental variables in our PCA analyses (Fig. 2), suggests that the resolution of our taxonomic annotations is sufficient to capture natural patterns within the creeks.

### Targeted analysis of denitrification genes.

Despite broad overall similarity between sediment communities in the two creeks, the abundance of nutrients in the enriched creek suggests that processes like denitrification have responded to the increased nutrient availability. We note that because our study did not directly measure denitrification, we cannot correlate an increase in genes related to dentrification to an increase in the biochemical process itself. However, previous data suggest that salt marsh sediments are limited by nitrate availability and that denitrification rates increase during experimental addition of nitrate (4, 37–40). Our observation that nutrient enrichment has led to a greater abundance of functional annotations related to denitrification (Fig. 3 and Table 1; see also Fig. S3 in the supplemental material) supports the hypothesis of a nutrient-induced increase in denitrifying bacteria and may provide a mechanism for increased denitrification rates.

Targeted analyses of annotation subgroups in this study identified a clear increase in the frequency of genes involved in denitrification in the enriched creek (Fig. 3). This effect was observed across sampling locations, suggesting a creek-wide effect of nutrient addition due to the sewage effluent. These results suggest that increased rates of denitrification in nutrient-enriched sediments (4, 41) may be partially explained by an increased abundance of denitrifying bacteria. In both creeks, several denitrification genes (notably respiratory nitrate reductase and copper-containing nitrite reductase genes) decreased in relative abundance at more-saline sites away from the headwaters (Fig. 3); this may result from the increased salinity, which has been shown previously to reduce rates of denitrification (40, 42, 43).

Our comparison of only two creeks raises the possibility that the observed differences in our samples are due to natural variation rather than to a direct effect of nutrient enrichment. While we recognize the possibility that unmeasured variables may account for differences found between these two creeks, and that strong differences in the frequency of some annotations are expected by chance, several aspects of our experimental design and results allow us to make strong conclusions about this system. First, whole groups of annotations corresponding to each of the biochemical pathways of denitrification were found to be increased in the enriched creek, which is highly unlikely by chance. Second, agnostic searches across the entire annotation string data set recovered enzymes in the denitrification pathway that were some of the most highly differentiated between the two creeks, representing 7 of the 11 most significantly divergent annotation groups between creeks (see Data set S3 and Fig. S3 in the supplemental material). Finally, we observed a gradient in the metagenomic reads related to each step in denitrification, despite inclusion of creek as a factor in the model (Table 1), which corresponds to the enrichment gradient as captured by % C in sediment. These results suggest that creek variation alone cannot account for differences in the abundance of denitrification annotations between the creeks.

### Agnostic search reveals other putative divergences in microbial functions.

Searches for highly divergent annotation subgroups across the entire data set between the two creeks identified denitrification-related subgroups as highly divergent, thereby recovering our hypothesis-based test for differences in the abundance of denitrification genes. This approach also identified strong differences in annotations related to heavy metal resistance, organic solvent resistance, osmotic stress, and phage resistance (see Fig. S4 and Data set S3 in the supplemental material), suggesting that stress and phage resistance functions may have also been influenced by exposure to sewage effluent. While large differences in annotation frequencies are expected by chance, we observed a consistent increase across a large number of annotations associated with each of these processes (see Fig. S3 in the supplemental material). These differences could have been related to effects of the sewage effluent that were unrelated to nutrient addition. For example, heavy metal contamination in wastewater is a well-known challenge (44), and our results suggest that microbial communities may respond through an increase in heavy metal resistance functions. Similarly, solvents used in household cleaners could be responsible for the abundance of organic solvent and osmoprotection annotations in the enriched creek. The possible reasons for a strong divergence in annotations related to phage resistance are less obvious but may warrant further research, as the divergence might imply differing host-parasite dynamics in response to the sewage effluent.

### Functional response of *nosZ* is taxonomically independent.

To gain insight into the taxonomic breadth over which microbial communities respond to denitrification, we examined the taxonomic distribution of a single gene, that encoding nitrous oxide reductase (*nosZ*), in both the reference and nutrient-enriched creeks. We chose to investigate *nosZ*, as it is broadly distributed phylogenetically and completes the denitrification process by reducing nitrous oxide, a potent greenhouse gas, to atmospheric nitrogen (45, 46). To do this, we assigned *nosZ* reads in our metagenomic data set to taxonomic groups based on homology to characterized *nosZ* sequences from the NCBI (see Materials and Methods). As with other denitrification-related annotation groups, our analysis showed that *nosZ* has greater frequency in the enriched creek (Table 1). However, we also found that the increased frequency of *nosZ* in the enriched creek does not correspond to the increased abundance of a single group of bacteria but instead is spread across a taxonomically diverse group of bacteria that all harbor the *nosZ* gene (Fig. 4; see also Fig. S5 in the supplemental material). With a few exceptions, different taxonomic *nosZ* variants tended to increase in proportion to their abundances in the reference creek (see Fig. S5). In other words, the relative frequency of each taxonomic *nosZ* variant was similar between the two creeks, despite an overall increase in the frequency of *nosZ* among all annotations in the enriched creek (see Fig. S5). This suggests that the microbial community response to anthropogenic nutrient addition may be independent of taxonomy.

Last, two recent papers identified a novel clade of *nosZ* (17, 18) and suggested that its prevalence in the environment may be underappreciated. Our analysis mapped ∼72% of *nosZ* annotations to this previously uncharacterized clade, very similar to the ∼70% of atypical *nosZ* recently reported by Orellana et al. (19) for agricultural soils in the U.S. corn belt. While additional studies are needed to establish whether this novel *nosZ* clade is functionally equivalent to that previously reported, our results suggest that it could play a primary role in environmental denitrification.

### Conclusions.

When we compared microbial communities receiving high rates of nutrient loading to those from reference communities across similar environmental gradients, we found very little difference in the overall community structures. In contrast, we found a clear fine-scale increase in functional genes associated with denitrification in a nutrient-enriched creek compared to a reference creek. Our results highlight a potential challenge for detecting such differences among similar environmental samples using taxonomic data alone. As we showed from our analysis of *nosZ*, a single functional gene can be increased across a wide range of functionally redundant taxa, thus reducing any taxonomic signal (Fig. 4). Future research that explicitly links taxonomy with functional genes underlying biogeochemical processes and gene expression through sequencing of mRNA will provide novel insights into microbial community responses to anthropogenic change. Further, such studies will help to further elucidate the ecological mechanisms of microbial community assemblage that are responsible for the apparent discrepancy between taxonomic and functional responses to environmental disturbance.

## Supplementary Material

Supplemental material
