# Supplementary material for: Functional Responses of Salt Marsh Microbial Communities to Long-Term Nutrient Enrichment
Source: Appl Environ Microbiol. 2016 Apr 18;82(9):2862–71. doi: 10.1128/AEM.03990-15 (PMC4836423; doi:10.1128/AEM.03990-15)
Supplement: Supplemental material [file supp_82_9_2862__index.html]

Supplemental material 

# Functional Responses of Salt Marsh Microbial Communities to Long-Term Nutrient Enrichment

## Supplemental material

- Supplemental file 1 -

  Locations of study creeks (Fig. S1); most-abundant taxa by genus according to MG-RAST annotations, and subsystems-level functional annotations (Fig. S2); select nitrogen-related annotation groups identified by agnostic word searches (Fig. S3); selected additional annotation groups identified by agnostic word searches that vary significantly between the enriched and reference creek (Fig. S4); relative frequencies of *nosZ* variants for the 20 most-abundant taxonomic clusters in each creek (Fig. S5); principal-component analysis of genus-level taxonomic annotations and subsystems-level functional annotations between each site and creek (Fig. S6).

  PDF, 2.4M
- Supplemental file 2 -

  Sample metadata, including GPS coordinates and physical and chemical properties, for water and sediments (Data Set S1).

  XLSX, 57K
- Supplemental file 3 -

  NCBI reference NosZ amino acid sequences, reference numbers, and isolate genera used for homology-based taxonomic assignments (Data Set S2).

  XLSX, 141K
- Supplemental file 4 -

  Most highly divergent annotation groups among enriched and reference creeks, identified by agnostic string-based annotation searches (Data Set S3).

  XLSX, 69K
