# Supplementary material for: Functional Responses of Salt Marsh Microbial Communities to Long-Term Nutrient Enrichment
Source: Appl Environ Microbiol. 2016 Apr 18;82(9):2862–71. doi: 10.1128/AEM.03990-15 (PMC4836423; doi:10.1128/AEM.03990-15)
Supplement: Supplemental material [file AEM.03990-15_zam999117120so1.pdf]

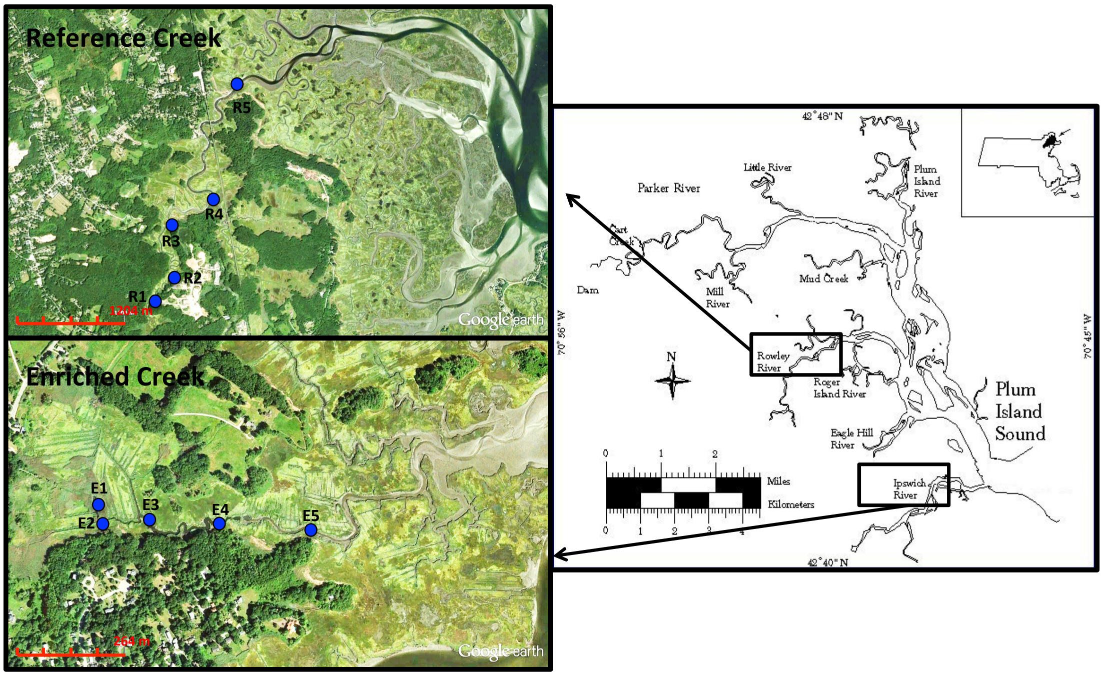

Figure S1. Locations of our study creeks. Sampling sites for enriched (Greenwood) creek and reference (Egypt) creek are shown in blue circles. The sewage outfall in the enriched creek is within 30 meters of head of the creek, with sample site E1 approximately 10 meters downstream of the sewage effluent.

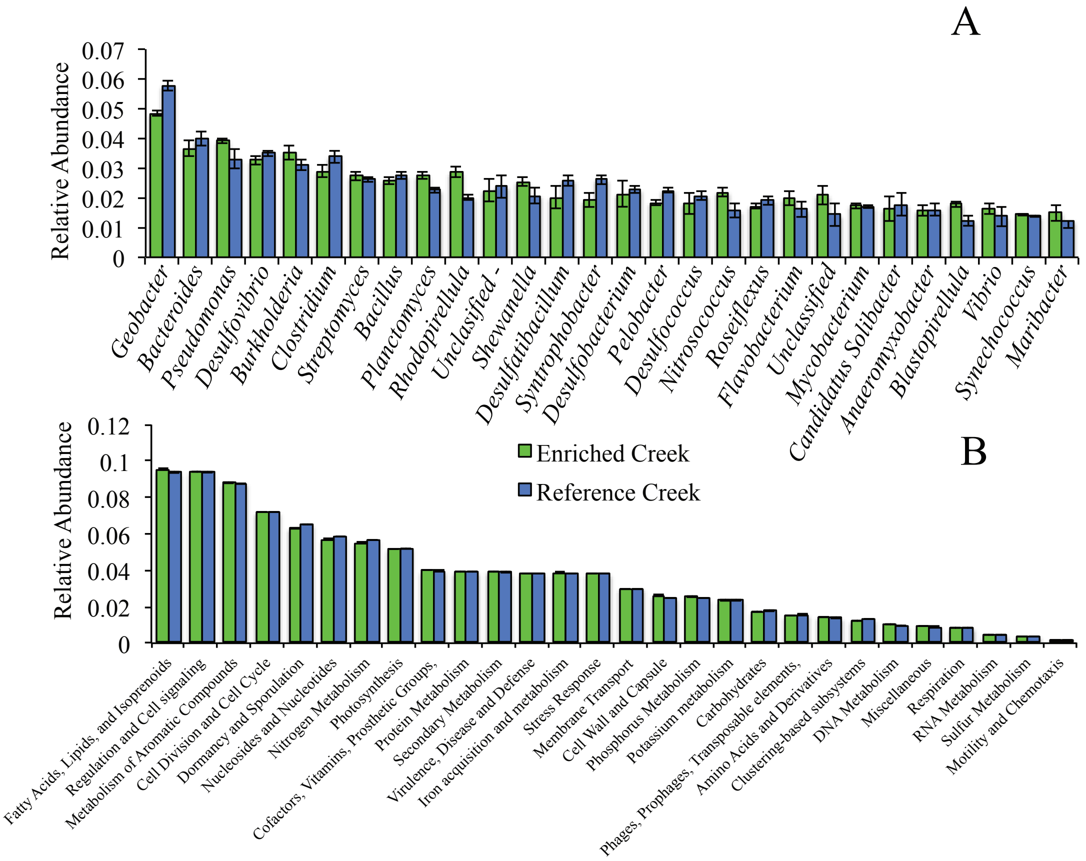

Figure S2. (**A**) Most abundant taxa by genus according to MG-RAST annotations, with frequencies for all sites averaged for each creek. Blue bars correspond to reference creek and green bars to enriched creek. (**B**) Subsystems level functional annotations, with blue bars indicating the average frequency at all sites in the reference creek, and green bars the average in the enriched creek.

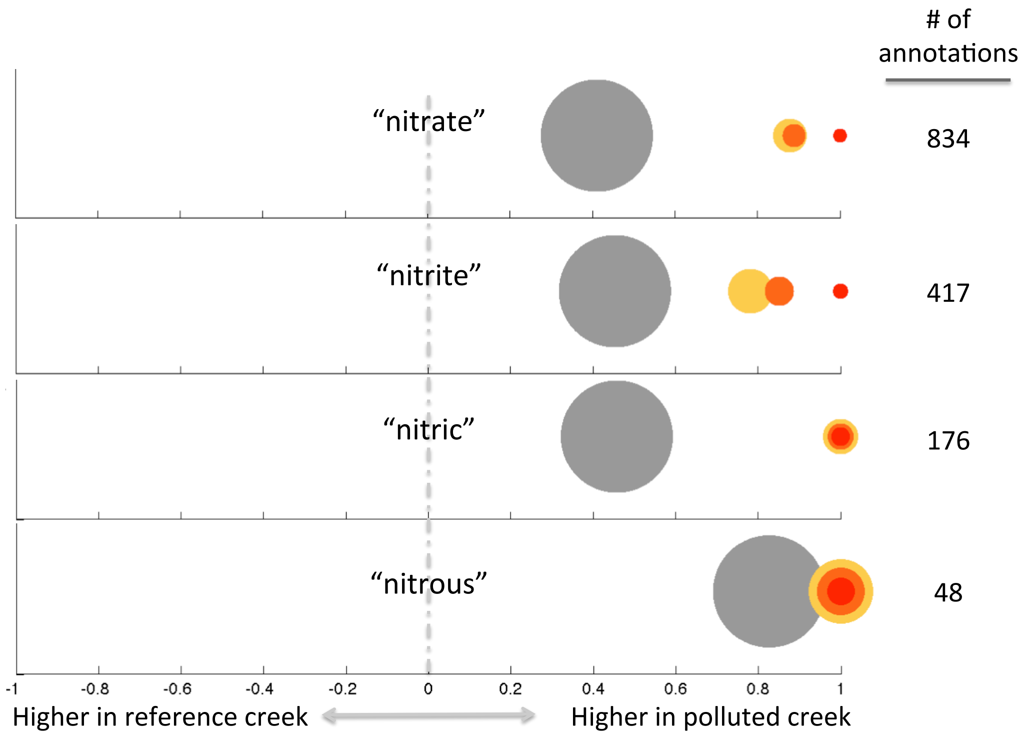

Figure S3. Select nitrogen-related annotation groups identified by agnostic word searches, with bubble sizes corresponding to the number of annotations in each Effect Level grouping: red bubbles are the highest Effect Level 3; orange are Level 2, yellow are Level 1; and grey are Level 0, i.e., all annotations in the group (see Experimental Procedures). Bubble sizes are normalized by all annotations for a gene family, so that grey bubble size is standard for all plots. Placement along the x-axis corresponds to the ratio of individual annotations with higher frequency in enriched vs. reference creek (see Annotation Ratio defined in Experimental Procedures).

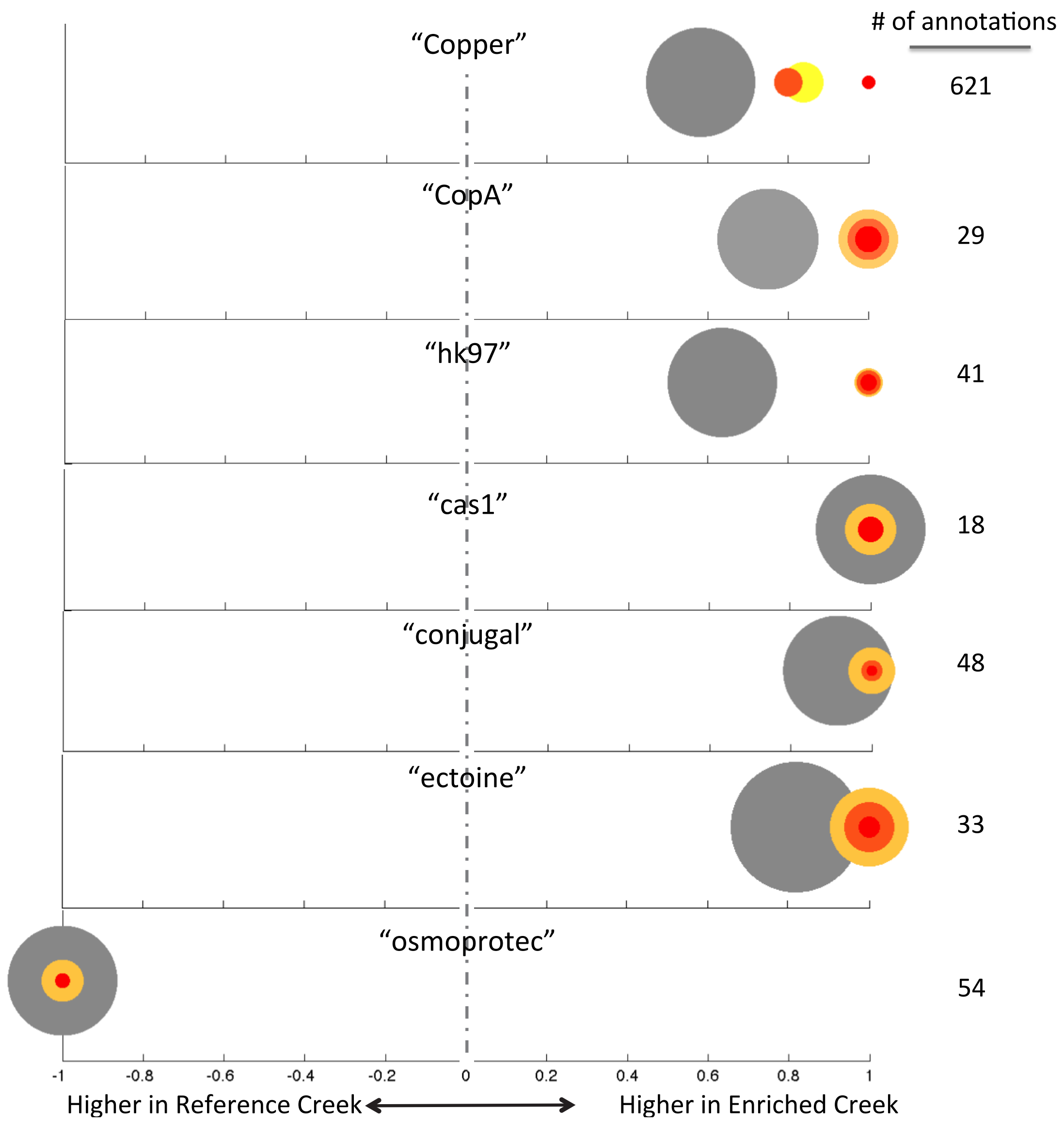

Figure S4. Selected additional annotation groups identified by agnostic word searches that vary significantly between the enriched and reference creek. Bubble sizes and colors corresponding to effect level groupings are as described in Fig. S3.

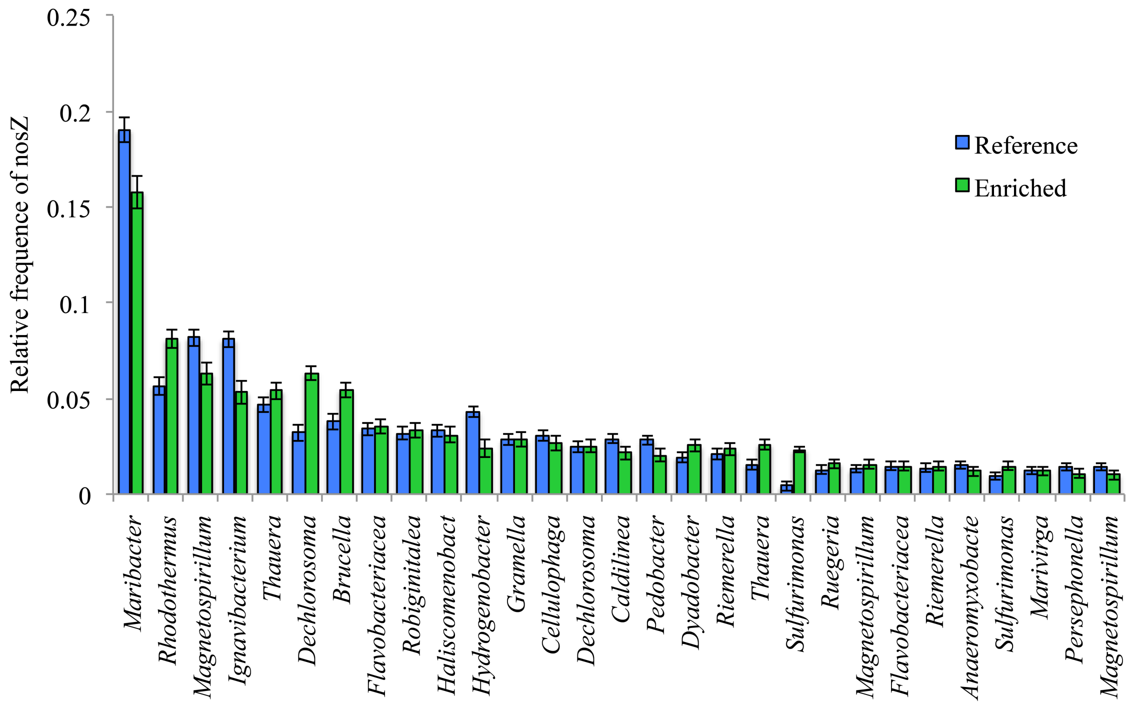

Figure S5. Relative frequency of nosZ variants for the 20 most abundant taxonomic clusters in each creek. Genera on the x axis represent taxonomically characterized nosZ variants to which our metagenomics reads mapped at >93% similarity. The higher abundance of nitrous oxide reductase annotations in the enriched creek is spread over a taxonomically diverse set of nosZ variants, with the two creeks maintaining a similar distribution of nosZ variants. Separate nosZ allelic variants mapping to the same genus are included in the plot.

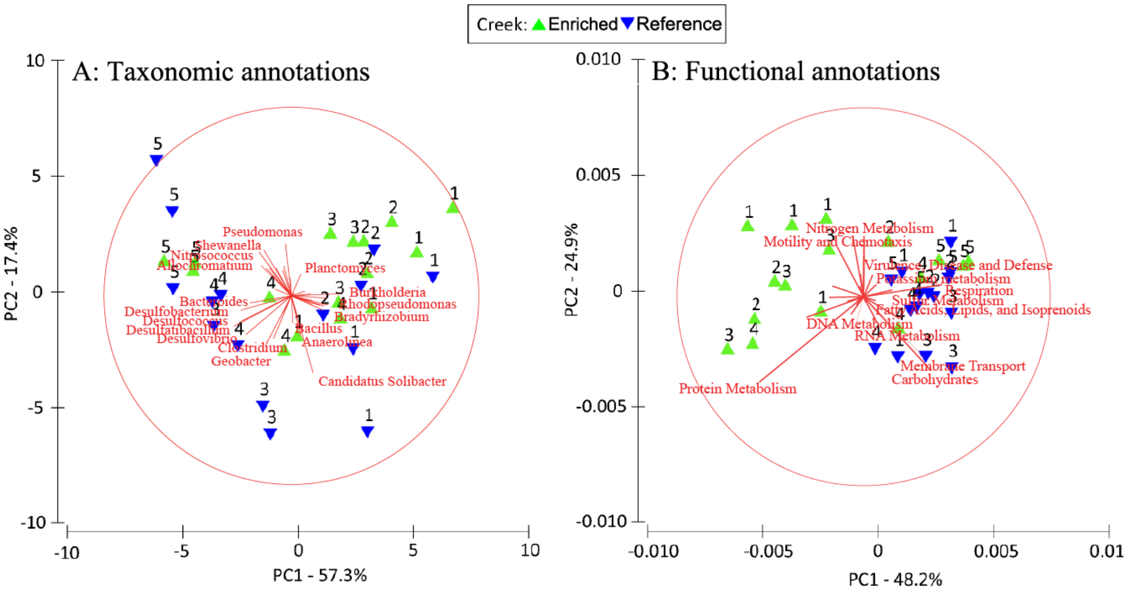

Figure S6. Principal component analysis of genus level taxonomic annotations (**A**) and subsystems level functional annotations (**B**) between each site and creek. Vector variables are plotted using PrimerE v6, note not all vector variables are labeled.
